# Physicochemical, Bioactive, and Clinical Evaluation of Blueberry‐Enriched Kombucha as a Functional Beverage

**DOI:** 10.1111/1750-3841.71337

**Published:** 2026-09-18

**Authors:** Angelica Morgan Anselmini, Gabrieli Lorandi, Marcieli Peruzzolo, Itamar Luís Gonçalves, Rogério Luis Cansian, Jamile Zeni, Eunice Valduga, André Keng Wei Hsu, Vivian Polachini Skzypek Zanardo, Geciane Toniazzo Backes

**Affiliations:** ^1^ Department of Food Engineering URI Erechim Erechim Brazil

## Abstract

This study aimed to develop a blueberry‐flavored kombucha and evaluate its functional properties and effects on human intestinal health. The beverage was produced from green tea and sucrose, fermented with a symbiotic culture of bacteria and yeasts (SCOBY), followed by the addition of blueberry syrup and a second fermentation stage. Active formulations (with viable microbiota) and sterilized control formulations were prepared. Physicochemical, microbiological, and bioactive analyses were performed, along with a controlled clinical trial involving 39 adults who consumed 100 mL of the beverage daily for 28 days. The active kombucha exhibited high microbial viability, with appropriate levels of yeasts, lactic acid bacteria, and acetic acid bacteria, as well as a low pH and safe alcohol content (0.20%). It also presented higher antioxidant activity (IC_50_ = 10.66 µL/mL) and elevated levels of phenolic compounds, flavonoids, and anthocyanins compared to the control. Clinically, participants consuming the active kombucha were associated with improvements in intestinal function, including normalization of stool consistency (Bristol scale type 4) and reductions in self‐reported abdominal symptoms such as bloating and pain. Approximately 90% of participants reported a perceived improvement in bowel function. The results suggest that blueberry kombucha is a promising functional beverage with favorable bioactive characteristics and the potential to support gastrointestinal health. However, these findings should be interpreted with caution due to the exploratory nature of the clinical evaluation and warrant confirmation in larger clinical studies involving clinically characterized populations.

**Practical Applications:**

This research can be applied by the food industry to create a ready‐to‐drink kombucha enriched with blueberry, combining good taste with ingredients that have the potential to support digestive health. It offers consumers a natural beverage option with antioxidant compounds and live cultures that may help maintain regular intestinal function. This product could be positioned as a functional drink for everyday consumption as part of a balanced diet.

## 1 Introduction

The understanding of the relationship between diet and intestinal health has advanced significantly in recent years, particularly with the recognition that the gut microbiota plays a fundamental role in digestion, metabolism, immune function, and the regulation of systemic inflammatory balance. Alterations in this microbiota are associated with disorders such as functional constipation, dysbiosis, and irritable bowel syndrome (IBS), which affect approximately 10%–20% of the global population, directly impacting quality of life and overall well‐being (Costa et al. 2025; Weber et al. 2022). In this context, functional foods containing viable microorganisms have gained increasing attention as non‐pharmacological strategies for improving gastrointestinal health and promoting intestinal homeostasis.

Among these foods, kombucha stands out as a fermented beverage produced through the symbiotic activity of bacteria and yeasts acting on tea infusions and sucrose substrates. The fermentation process promotes the formation of organic acids, vitamins, and antioxidant compounds, while preserving viable microorganisms capable of modulating the gut microbiota (Batista et al. 2023; Sanwal et al. 2023). Recent studies have demonstrated that regular consumption of kombucha may improve intestinal motility, reduce abdominal discomfort, and contribute to microbial rebalancing, reinforcing its potential as a functional beverage with benefits for gastrointestinal health (Taupiqurrohman et al. 2024; Costa et al. 2025).

In parallel, fruits rich in bioactive compounds such as anthocyanins, flavonoids, and phenolic compounds have been increasingly explored for the enrichment of functional beverages. Blueberry (*Vaccinium myrtillus*) is widely recognized for its high antioxidant capacity, exhibiting protective effects against oxidative stress and inflammatory processes, as well as contributing to the maintenance of intestinal and metabolic health (Zhang et al. 2025; R. M. Ahmed et al. 2023). The combination of fermented beverages with fruits rich in phenolic compounds has been identified as a promising strategy to enhance functional benefits and improve the stability of bioactive compounds (Balmaseda et al. 2024; Barbosa et al. 2022).

Despite the growing interest in kombucha and its physiological effects, there remains a scarcity of clinical studies investigating its direct influence on intestinal function, particularly when combined with bioactive fruits. The impact of blueberry addition on the microbiological stability and antioxidant profile of kombucha is still poorly explored, despite evidence that fruit phenolic compounds may interact with fermentation‐derived metabolites. Furthermore, there is a substantial gap in comparative investigations evaluating the effects of live (active) kombucha versus sterilized kombucha on intestinal health, which is essential to distinguish benefits attributable to viable microbiota from those derived from stable metabolites. In this context, the present study demonstrates an innovative approach by developing and characterizing a blueberry‐flavored kombucha, assessing not only its physicochemical and bioactive parameters but also its potential effects on human intestinal health through a controlled clinical trial.

## 2 Materials and Methods

### 2.1 Material

The symbiotic culture of bacteria and yeasts (SCOBY) used in the production of kombucha was obtained from a certified supplier (Culturas Probióticas, Brazil). The microbial composition of the SCOBY was not identified at the strain or species level prior to the study. Therefore, the microbiological evaluation focused on the quantification of viable groups commonly associated with kombucha fermentation, including yeasts, lactic acid bacteria, and acetic acid bacteria.

The green tea (*Camellia sinensis*) used in the preparation of the beverage's base infusion, as well as the blueberry fruits (*V. myrtillus* L.), was purchased from the local market. The fruits were acquired between November 2024 and January 2025, corresponding to the regional harvest season.

After acquisition, the blueberry fruits were subjected to manual sorting, and immature, damaged, or deteriorated units were discarded. The selected fruits were washed under running water and subsequently sanitized in a sodium hypochlorite (NaClO) solution at 200 ppm for 30 min, according to the procedure described by Gil et al. (2009). Thereafter, the fruits were rinsed with potable water, drained, and packed in previously sanitized and properly identified polyethylene containers. The samples were stored under freezing conditions at −10°C until use.

### 2.2 Preparation and Characterization of Blueberry‐Flavored Kombucha

For fermentation, an infusion was prepared using 5 g/L of dried green tea leaves (*Camellia sinensis*) and water previously heated to 90°C for 5 min. The infusion was then filtered, supplemented with 50 g/L of sucrose, and inoculated with the kombucha culture containing approximately 18 g/L (w/w) of SCOBY. The fermentation vessel was covered with gauze to allow aerobic fermentation and incubated at 25°C for 7 days without agitation (Jayabalan et al. 2014).

At the end of fermentation, the beverage was filtered through a sterile sieve to remove the SCOBY, and 10% (v/v) of syrup prepared with blueberries was added. For the preparation of the syrup, 120 g/L of sucrose and 500 g/L of whole blueberry fruits were used. This mixture was boiled for 30 min, filtered, and cooled. The kombucha mixed with the blueberry syrup was then incubated at 25°C for an additional 3 days to allow the carbonation process.

A fraction of the kombucha was maintained without the addition of blueberry syrup in order to evaluate the bioactive properties of kombucha without blueberry supplementation; this fraction was also incubated for 3 days at 25°C for carbonation. These formulations were designated as active kombuchas, with or without blueberry addition.

The control kombucha formulations were prepared under the same conditions, with and without the addition of blueberry syrup; however, at the end of the fermentation process, they were sterilized at 121°C for 15 min to eliminate viable microorganisms and interrupt fermentative activity. Autoclaving was intentionally used to obtain a microbiologically inactive control beverage, allowing a clearer distinction between the effects associated with live microbial communities and those related to stable fermentation‐derived metabolites. The formulations were then bottled in 400 mL polyethylene terephthalate (PET) bottles and stored under refrigeration (5°C) until the analyses were performed (6 days) and subsequently delivered to the evaluators for the clinical trial. All formulations were prepared and analyzed in triplicate.

For the characterization of the kombucha samples, physicochemical analyses were performed, including instrumental color, pH, acidity, and alcohol content. To evaluate microbial viability, analyses were conducted to determine yeast, lactic acid bacteria, and acetic acid bacteria counts. Bioactive properties were assessed through the quantification of phenolic compounds, flavonoids, anthocyanins, and antioxidant activity. The clinical trial was conducted using the Bristol Stool Scale and the IBS‐SSS (Irritable Bowel Syndrome Severity Scoring System). The project was approved by the Research Ethics Committee (URI‐Erechim) and registered on Plataforma Brazil under CAAE number 39470820.0.0000.5351.

### 2.3 Physicochemical Analyses

#### 2.3.1 Acidity

Acidity was determined by titration using standardized 0.01 N NaOH and phenolphthalein as an indicator. Briefly, 10 mL of sample was diluted in distilled water and titrated until the appearance of a persistent light pink coloration. Results were expressed as % (w/v) of acetic acid.

#### 2.3.2 pH

pH was measured using a pH meter (Digimed, model DM‐22). Calibration was performed using standard buffer solutions at pH 4.0 and 7.0 prior to analysis.

#### 2.3.3 Alcohol Content

Alcohol content was determined by distillation of the fermented beverage followed by density measurement of the distillate at 20°C using an alcoholometric conversion table, according to Li et al. (2021). Briefly, 100 mL of kombucha sample was distilled, and the ethanol concentration was calculated from the relative density of the recovered distillate and expressed as % (v/v). Measurement was performed according to standard analytical methods.

#### 2.3.4 Instrumental Color

Color was determined using a colorimeter (Minolta Chroma Meter, model CR‐400) in the CIELAB color system, based on the three components: L* (lightness or brightness), ranging from 0 (black) to 100 (white), and the chromaticity coordinates a* and b*, which vary from −a* (green) to +a* (red) and from −b* (blue) to +b* (yellow), respectively. Measurements were performed at room temperature (25°C ± 2°C) directly on the sample surface.

#### 2.3.5 Soluble Solids (°Brix) and Fermentation Kinetics

The soluble solids content of the final kombucha formulations and blueberry syrup was determined in triplicate using an Abbe refractometer (Biobrix WYA‐2WAJ‐DIGI, Biobrix, Brazil), with a measurement range of 0–95 °Brix and an accuracy of ±0.1 °Brix. Before analysis, the instrument was calibrated with distilled water, and measurements were performed at 20°C to ensure analytical accuracy. Approximately 0.3 mL of each homogenized sample was placed directly on the prism, and the readings were expressed as °Brix.

### 2.4 Determination of Bioactive Compounds and Antioxidant Activity

#### 2.4.1 Total Phenolic Compounds

Total phenolic compounds were determined using the Folin–Ciocalteu colorimetric method according to Singleton et al. (1999). Aliquots of the samples were mixed with Folin–Ciocalteu reagent and sodium carbonate solution, followed by incubation in the dark at room temperature for 30 min. Absorbance was measured at 765 nm using a UV–Vis spectrophotometer. Gallic acid was used to construct the calibration curve, and the results were expressed as milligrams of gallic acid equivalents per 100 g of extract (mg GAE/100 g).

#### 2.4.2 Flavonoids

Flavonoids were determined according to the method described by Garrido et al. (2013). Sample extracts were mixed with aluminum chloride reagent and maintained at room temperature for color development. Absorbance was measured at 415 nm using a UV–Vis spectrophotometer. Results were expressed as milligrams of quercetin equivalents per 100 g of extract (mg QE/100 g).

#### 2.4.3 Anthocyanins

Anthocyanins were determined according to the method described by AOAC (2005). The extracts were diluted separately in potassium chloride buffer (pH 1.0) and sodium acetate buffer (pH 4.5), and absorbance readings were obtained at 520 and 700 nm using a UV–Vis spectrophotometer. Total monomeric anthocyanin content was calculated based on the difference in absorbance between the two pH conditions and expressed as malvidin‐3‐glucoside equivalents (mg/L).

#### 2.4.4 Antioxidant Activity

Antioxidant activity was evaluated using the DPPH and ABTS radical scavenging assays. For the DPPH method, antioxidant activity was determined by measuring the reduction in absorbance of the 2,2‐diphenyl‐1‐picrylhydrazyl radical at 515 nm after reaction with the sample extracts, according to Silvestri et al. (2010). Different extract concentrations were prepared, and the concentration required to inhibit 50% of the radicals (IC_50_) was calculated by linear regression. For the ABTS assay, the ABTS●+ radical solution was prepared according to Boroski et al. (2015), and absorbance was measured at 734 nm after reaction with the extracts. Results were expressed as IC_50_ and as millimoles of Trolox equivalents per gram of sample (mmol Trolox/g of sample).

### 2.5 Microbiological Analyses

To determine acetic acid bacteria, yeast mannitol peptone agar supplemented with 500 mg/L cycloheximide was used. Plates were incubated for 5 days at 28°C (Lončar et al. 2014). The viability of lactic acid bacteria was determined using MRS agar (de Man, Rogosa, and Sharpe) containing 500 mg/L cycloheximide, with plates incubated for 3 days at 37°C (Pereira et al. 2017). Viable yeast counts were determined on Sabouraud agar (4%) supplemented with 50 mg/L chloramphenicol, with plates incubated for 3 days at 28°C (Pereira et al. 2017). Results were expressed as log colony forming units (CFU) per milliliter. Microbiological analyses were performed on the 6th day and after 28 days of storage at 4°C to confirm the maintenance of cellular viability during the clinical trial period.

### 2.6 Clinical Trials

The study was designed as a randomized, controlled, quantitative clinical trial. The sample consisted of 39 participants, selected by convenience sampling and recruited through an open invitation to participate in the study, in accordance with the established inclusion criteria. Eligible participants were individuals aged 18–59 years, of both sexes, considered healthy, and who agreed to participate in the research. Exclusion criteria included individuals diagnosed with or under treatment for diabetes mellitus, arterial hypertension, dyslipidemia, chronic constipation, cancer, cognitive impairment, or presenting with intolerance or allergy.

Randomization was performed by an independent researcher using the Research Randomizer tool. Participants were allocated into two groups: the intervention group, which received blueberry kombucha containing live microbial communities, and the control group, which received sterilized kombucha. Each volunteer received seven 400‐mL bottles of the beverage for consumption throughout the clinical trial. The intake protocol was standardized at 100 mL per day for 28 consecutive days, preferably in the morning, before or after breakfast, with the beverage kept refrigerated at 5°C. Participants were instructed not to modify their dietary habits, physical activity routine, or medication use during the study period.

Participants completed a printed questionnaire containing sociodemographic data (age, sex, education level, and income) and information related to intestinal health. Intestinal transit was assessed using the Bristol Stool Form Scale, which classifies stool into seven types based on their characteristics, allowing the recording of the frequency of each type (Martinez and Azevedo 2012). In addition, the IBS‐SSS was applied as a symptom assessment tool, which evaluates abdominal pain and bloating, satisfaction with bowel habits, and the impact of symptoms on daily life (Ida et al. 2017). Both the Bristol Stool Form Scale and the IBS‐SSS were applied at the beginning and at the end of the kombucha consumption period (28 days).

### 2.7 Statistical Analysis

The results of the physicochemical and microbiological analyses were statistically analyzed using analysis of variance (ANOVA), and when significance was detected, the Tukey post hoc test was applied. All tests were performed at a significance level of 5% (*p* < 0.05). Clinical trial results for the Bristol Stool Scale were analyzed using the Friedman test, followed by Dunn's post hoc test and the Mann–Whitney test (*p* < 0.05).

## 3 Results and Discussion

### 3.1 Fermentation Kinetics

**FIGURE 1:**
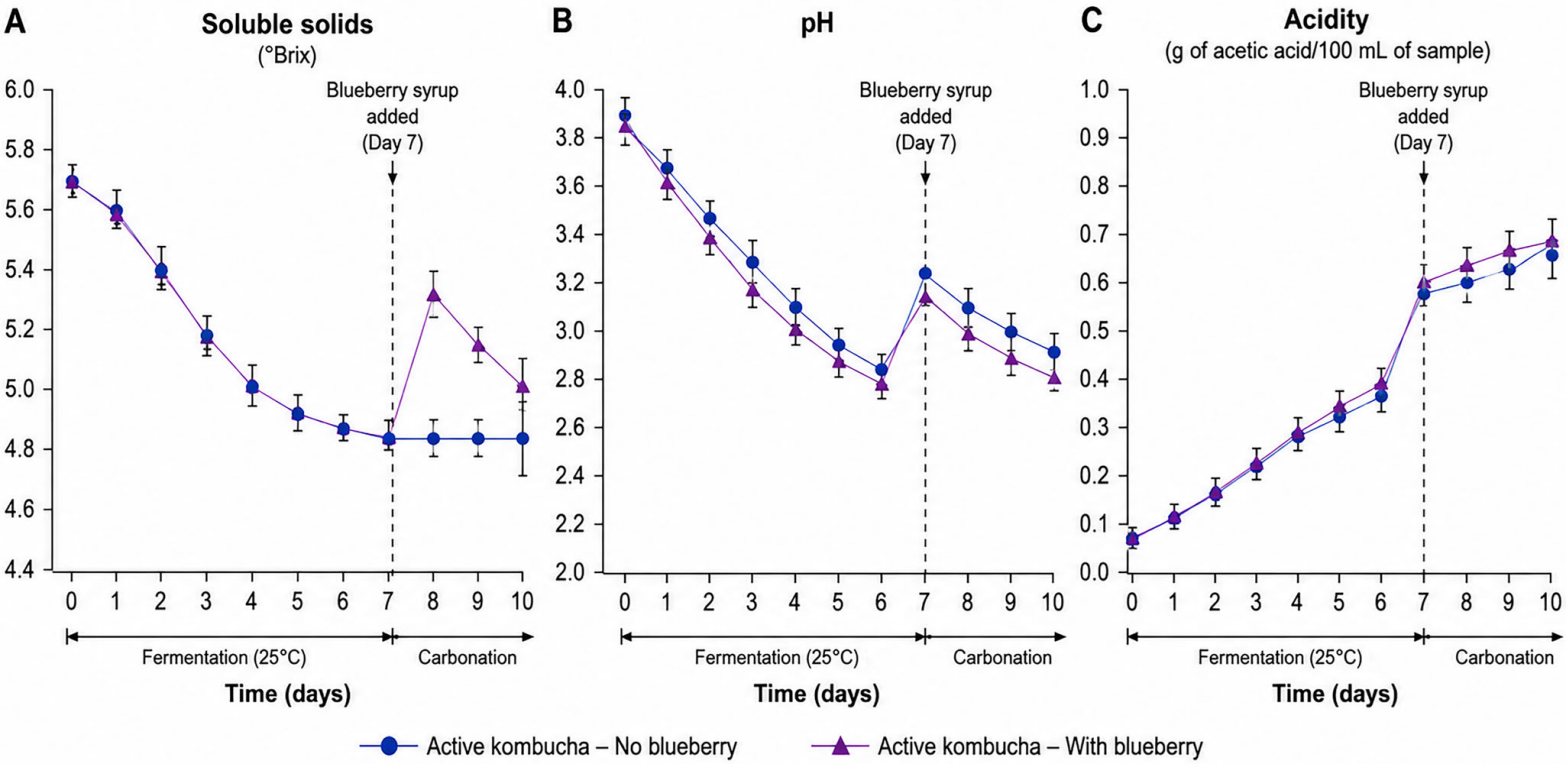
Changes in soluble solids (°Brix) (A), pH (B), and titratable acidity (g of acetic acid/100 mL of sample) (C) during the fermentation and carbonation of active kombucha prepared from green tea supplemented with 50 g/L sucrose, with or without the addition of 10% (v/v) blueberry syrup on Day 7.

The fermentation profile reconstructed from the experimentally determined soluble solids together with literature‐supported intermediate values is presented in Figure 1. During the primary fermentation (0–7 days), soluble solids gradually decreased from approximately 5.7 to 4.82 °Brix, reflecting the progressive consumption of fermentable sugars by the SCOBY consortium. This behavior is characteristic of kombucha fermentation, in which yeasts initially hydrolyze sucrose into glucose and fructose, followed by carbohydrate consumption for ethanol production, while acetic acid bacteria subsequently oxidize ethanol into organic acids.

Simultaneously, pH decreased continuously from approximately 3.9 to values below 3.2, accompanied by a progressive increase in titratable acidity. These changes reflect the accumulation of organic acids, mainly acetic acid, produced during fermentation.

On Day 7, the addition of 10% (v/v) blueberry syrup slightly increased the soluble solids because of the introduction of additional fermentable carbohydrates. During the subsequent 3‐day carbonation stage, soluble solids decreased again, whereas pH continued to decline and titratable acidity increased, reaching the experimentally determined final values shown in Table 1.

**TABLE 1:** Microbiological, physicochemical, and antioxidant characterization of active kombucha, control, and blueberry syrup.

Parameters		Active kombucha			Kombucha control		Syrup
		With blueberry		No blueberry	With blueberry	No blueberry	
Yeasts (log_10_ UFC/mL)	Initial	6.35 ± 0.37		6.32 ± 0.25	0	0	0
	28 days	5.87 ± 0.20		5.84 ± 0.26	0	0	0
Lactic acid bacteria (log_10_ UFC/mL)	Initial	4.60 ± 0.31		4.57 ± 0.27	0	0	0
	28 days	3.77 ± 0.15		3.75 ± 0.17	0	0	0
Acetic bacteria (log_10_ UFC/mL)	Initial	5.42 ± 0.23		5.40 ± 0.35	0	0	0
	28 days	5.35 ± 0.11		5.32 ± 0.21	0	0	0
pH		2.83^a^ ± 0.12		2.96^a^ ± 0.14	2.87^a^ ± 0.13	2.92^a^ ± 0.13	2.79^a^ ± 0.12
Acidity (g of acetic acid/100 mL of sample)		0.686^a^ ± 0.042		0.668^a^ ± 0.031	0.670^a^ ± 0.026	0.664^a^ ± 0.032	0.270^b^ ± 0.021
Alcohol content (%, v/v)		0.20^a^ ± 0.01		0.10^b^ ± 0.02	0^c^	0^c^	0^c^
Flavonoids (mg QE/L of kombucha)		52.29^b^ ± 0.06		29.34^d^ ± 0.22	54.41^b^ ± 0.01	35.46^c^ ± 0.33	140.39^a^ ± 0.60
Anthocyanins (mg malvidin‐3‐glucoside/L)		34.04^b^ ± 3.10		0^d^	0.413^c^ ± 0.03	0^d^	479.6^a^ ± 25.10
Phenolic compounds (mg GAE/L of kombucha)		523.08^b^ ± 40.19		323.94^c^ ± 30.59	467.31^b^ ± 53.39	383.27^c^ ± 43.39	1614^a^ ± 30.59
Antioxidant activity IC_50_ (µL of kombucha/mL)		10.66^c^ ± 1.02		25.33^a^ ± 2.54	16.00^b^ ± 1.70	23.54^a^ ± 1.03	3.70^d^ ± 1.50
Color parameters		L*	49.34^b^ ± 1.84	60.91^a^ ± 2.54	47.99^b^ ± 1.70	58.65^a^ ± 2.01	46.89^b^ ± 1.89
		a*	23.24^b^ ± 0.89	−0.96^c^ ± 0.01	21.76^b^ ± 0.96	−0.97^c^ ± 0.01	26.67^a^ ± 0.82
		b*	6.24^c^ ± 0.16	7.92^b^ ± 0.15	6.47^c^ ± 0.29	8.12^b^ ± 0.17	16.45^a^ ± 0.76

*Note*: Different lowercase letters on the same line show a significant difference (*p* < 0.05) according to Tukey's test.

The kinetic profile agrees with fermentation patterns commonly reported for green tea kombucha prepared with sucrose under similar fermentation conditions, characterized by progressive substrate consumption, a continuous decrease in pH, and the accumulation of organic acids throughout fermentation (Jayabalan et al. 2014; Lončar et al. 2014; Alves et al. 2025).

### 3.2 Microbiological Characterization of Kombucha

The active kombucha exhibited a microbial profile consistent with that widely reported in the literature, characterized by the predominance of yeasts and acetic acid bacteria, along with a lower proportion of lactic acid bacteria. This symbiotic consortium is responsible for producing metabolites that directly contribute to the beverage's functionality and stability (Wang et al. 2022). The reductions observed in yeast populations (∼0.5 log) and, particularly, in lactic acid bacteria (∼0.8 log) after 28 days are consistent with patterns commonly reported during the storage of fermented beverages. These findings highlight the greater sensitivity of lactic acid bacteria compared to acetic acid bacteria, which tend to remain more stable over time (Grassi et al. 2022).

The incorporation of blueberry did not significantly (*p* > 0.05) affect microbial viability, indicating the technological compatibility of the ingredient. This finding aligns with studies showing that the addition of fruits to kombucha can increase phenolic compounds and antioxidant activity, but does not necessarily reduce microbial viability (Barbosa et al. 2022). The complete absence of microorganisms in the autoclaved controls and in the syrup itself confirms both the effectiveness of the thermal treatment and the appropriate hygienic–sanitary conditions of the ingredient. Despite the known antimicrobial activity of blueberry phenolic compounds, the concentrations used were not sufficient to affect the established microbiota (R. A. Ahmed et al. 2024; Shen et al. 2014).

The results indicate that kombucha maintained functional microbial viability over the 28‐day period, with greater stability of acetic acid bacteria and expected reductions in yeast and lactic acid bacteria populations. The addition of blueberry enhanced the beverage's bioactive profile without compromising fermentation, while the controls confirmed the absence of external microbial interference. These findings reinforce the potential functional role of kombucha's characteristic microbiota, comprising yeasts, lactic acid bacteria, and acetic acid bacteria, which contributes to intestinal balance, strengthening of the mucosal barrier, immune modulation, pathogen inhibition, and the production of vitamins and short‐chain fatty acids (Batista et al. 2023). Although the counts observed were lower than those reported by Sadok et al. (2025), who found approximately 7.5 log_10_ CFU/mL after 6 days of fermentation, the results of the present study confirm the presence of an active and functional microbial consortium capable of generating beneficial metabolites and supporting a healthy gut microbiota (Yuan et al. 2022).

### 3.3 Physicochemical Characterization

The physicochemical parameters measured at the end of fermentation are presented in Table 1. The final soluble solids content (4.82 °Brix) indicates partial utilization of fermentable sugars, with residual soluble solids remaining in the beverage.

The active kombucha, in both the versions with and without blueberry, exhibited a low pH (∼2.83 ± 0.12 and 2.96 ± 0.14) (Table 1), indicating an acidic and mature fermentation. The intense acidification can be attributed to the production of organic acids, especially acetic acid, by the symbiotic microbial community present in the kombucha, in which acetic acid bacteria oxidize ethanol produced by yeasts to form acetic acid (Tran et al. 2020; Thinzar Aung 2022; Sadok et al. 2025).

The recorded titratable acidity (∼0.68 g of acetic acid per 100 mL) reinforces this acidic profile, reflecting the significant accumulation of acetic acid during the fermentation process. This type of accumulation is common in kombucha, as acetic acid is the main organic acid produced in the fermentative system. Related studies have reported increasing concentrations of organic acids, primarily driven by the activity of acetic acid bacteria, which contribute to the pH decrease throughout fermentation (Alves et al. 2025).

Regarding the alcohol content, 0.20% (v/v) was observed in the blueberry kombucha and 0.10% (v/v) in the version without blueberry, while no detectable alcohol was found in the autoclaved controls. These low ethanol levels are consistent with reports of controlled fermented kombuchas, in which yeasts initially produce ethanol, but part of it is promptly metabolized by acetic acid bacteria to form acetic acid (Tran et al. 2020). This difference is associated with the presence of the blueberry‐flavored sugar syrup, which provided additional fermentable substrates, favoring ethanol production by the yeasts in the symbiotic culture (SCOBY). In the control kombuchas, which were fermented and subsequently autoclaved in an open container, the alcohol was completely eliminated, explaining the absence of ethanol in these samples. Even with the addition of blueberry, the alcohol content remained below 0.5% (v/v), keeping the formulations within the classification of nonalcoholic beverages. In Brazil and the United States, for a beverage to be considered nonalcoholic, the ethanol concentration must be below 0.5%; in contrast, in Italy and France, this threshold is below 1.2% (MAPA 2019; Fabricio et al. 2023).

From a safety and stability perspective, the extremely low final pH promotes the inhibition of undesirable microorganisms, contributing to the preservation of the beverage during storage. This acidic control is considered essential for the microbiological safety of kombucha (Jayabalan et al. 2014; Alves et al. 2025).

Furthermore, the results clearly reflect the classic microbial dynamics of kombucha: the interaction between yeasts and acetic acid bacteria is essential for shaping the final chemical profile of the beverage, with acetic acid bacteria converting ethanol into acetic acid, thereby contributing to the residual acidity and low pH (Zou et al. 2023).

### 3.4 Bioactive Compounds and Antioxidant Activity

The results presented in Table 1 indicate that the blueberry‐enriched kombucha shows significantly higher concentrations of flavonoids (52.29 mg QE/L) and anthocyanins (34.04 mg/L) (*p* < 0.05) compared to the kombucha without fruit, in addition to an elevated total phenolic content (523.08 mg GAE/L). This increase in polyphenolic compounds was associated with enhanced antioxidant potential, as reflected by the lower IC_50_ value (10.66 µL/mL). However, although anthocyanins showed substantial reduction after sterilization, total phenolic compound values remained relatively stable. This behavior may be related to the differential thermal stability of phenolic subclasses during processing, since different classes of phenolic compounds exhibit distinct oxidation susceptibilities. In addition, the Folin–Ciocalteu assay estimates the overall reducing capacity of the sample, which may contribute to the apparent maintenance of total phenolic values even after degradation of thermolabile compounds such as anthocyanins (Sui et al. 2016; Rocha‐Guzmán et al. 2023). These findings reinforce that different classes of bioactive compounds present distinct sensitivities to thermal processing.

The blueberry syrup, in turn, exhibited even higher concentrations of flavonoids (140.39 mg QE/L), anthocyanins (479.6 mg/L), and total phenolics (1614 mg GAE/L), which translates into substantially greater antioxidant activity (IC_50_ of 3.70 µL/mL).

These results are consistent with the kombucha literature, which shows that the addition of functional ingredients such as fruits or plants tends to increase the levels of polyphenols and flavonoids, thereby enhancing the beverage's antioxidant capacity (Jakubczyk et al. 2025). In kombuchas fermented from tea, particularly green tea, substantial amounts of flavonoids and phenolic acids have been identified and strongly correlated with high antioxidant activity, confirming the importance of these compounds for the beverage's functional properties (Lacerda et al. 2025). The addition of blueberry to kombucha not only increases the content of bioactive compounds but also enhances the beverage's antioxidant efficacy, without compromising microbial viability or physicochemical stability. These findings are promising for the development of functional kombuchas with higher nutritional value and potential therapeutic applications.

### 3.5 Instrumental Color Parameters

The variation in the color parameters of kombucha with and without blueberry (Table 1) directly reflects the stability of phenolic compounds, particularly anthocyanins, which are highly sensitive to changes in pH, oxygen, and enzymatic activity. The observed reduction in L* values indicates a progressive darkening, a typical phenomenon in anthocyanin‐rich matrices subjected to fermentation, resulting both from pigment degradation and from the formation of secondary compounds derived from oxidative reactions and condensation processes (Sui et al. 2016).

The increase in a* values suggests an intensification of the red coloration, consistent with the release and transformation of anthocyanins during the microbial metabolism of sugars. Similar results have been reported in kombuchas produced with maqui juice, where fermentation promoted structural changes in anthocyanins, altering the chromatic profile and directly contributing to the increase in antioxidant activity (Rocha‐Guzmán et al. 2023). Similarly, studies using microcapsules of pigmented extracts show that small structural changes in anthocyanins can lead to marked variations in red hue, reinforcing the sensitivity of this parameter (Romero‐González et al. 2020).

The decrease in b* values suggests a loss of the yellow component, a behavior consistent with the degradation of glycosylated flavonoids in acidic environments, as observed in fruit‐based fermented beverages enriched with anthocyanins (Cánovas et al. 2025).

These results confirm that the L*, a*, and b* parameters are sensitive indicators of the biochemical transformations that occur during fermentation, reflecting both the degradation and the formation of new pigments derived from phenolic compounds. Moreover, they reinforce the close relationship between color changes, the structural stability of anthocyanins, and antioxidant potential, in agreement with what has been reported for kombuchas formulated with fruits rich in bioactive pigments.

### 3.6 Clinical Trial

The group of participants in the clinical trial involving blueberry‐flavored kombucha was composed predominantly of women (95%), with ages ranging from 18 to over 50 years, mostly concentrated in the 35–50 age range. A high level of education was observed, with 46% having completed higher education and 18% holding postgraduate degrees, reflecting a profile with good access to information and potentially greater awareness regarding health and well‐being. Regarding body mass index (BMI), although some individuals were classified as overweight or as having class I or II obesity, the majority (54%) fell within the normal weight range.

#### 3.6.1 Initial Profile of Intestinal and Dietary Habits

**FIGURE 2:**
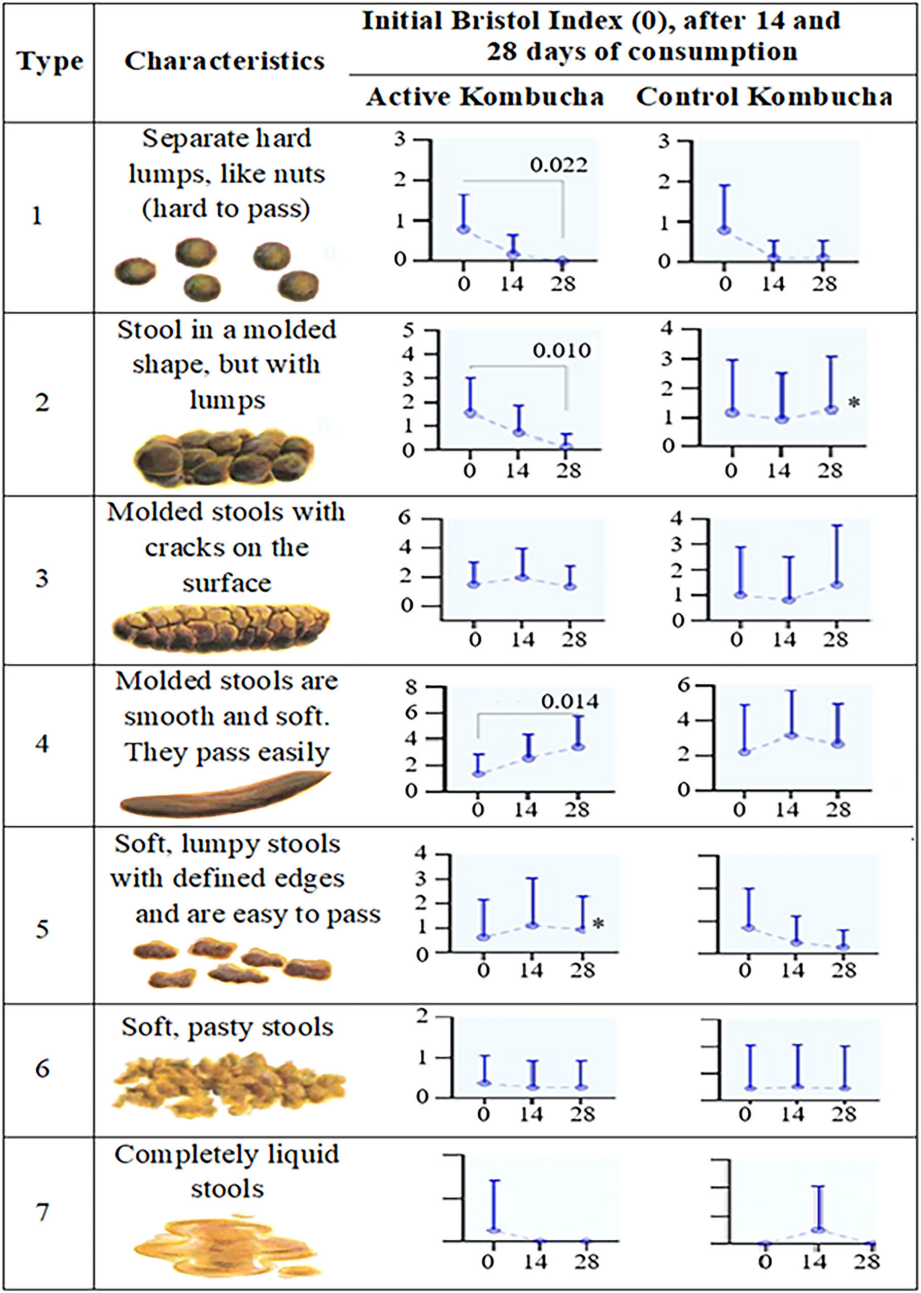
Initial profile of intestinal and dietary habits of the clinical trial participants.

The initial profile data on intestinal and dietary habits (Figure 2) indicate that only 13% of participants reported using laxatives, a value consistent with the prevalence observed among adults in Western countries (1%–18%) and in older adults (10%) (Werth and Christopher 2021). On the other hand, 23% reported using leaves or herbs, and 49% stated that they modify their diet, either by consuming or restricting certain foods, to improve intestinal function. These results suggest a group with conscious eating habits aimed at intestinal regulation, although population data on the use of herbal products and dietary adjustments are still limited.

Regarding gastrointestinal symptoms, 15% reported pain during defecation and 49% mentioned flatulence, indicating mild intestinal discomfort in a portion of the participants. Functional symptoms such as bloating and gas are common in the population, occurring weekly in approximately 17%–20% of adults (Ballou et al. 2023), with gas elimination between 10 and 20 times per day being considered physiological (Hasler 2006). Thus, the high reported incidence of flatulence may reflect greater attention to intestinal function or increased individual sensitivity to gastrointestinal symptoms.

Finally, 87% of participants reported not consuming kombucha, indicating that this beverage is not yet part of the regular habits of this group, a finding consistent with observations that kombucha consumption is still an emerging practice in Brazil (Dartora et al. 2023).

#### 3.6.2 Bristol Stool Scale: Stool Consistency According to the Bristol Stool Scale

**FIGURE 3:**
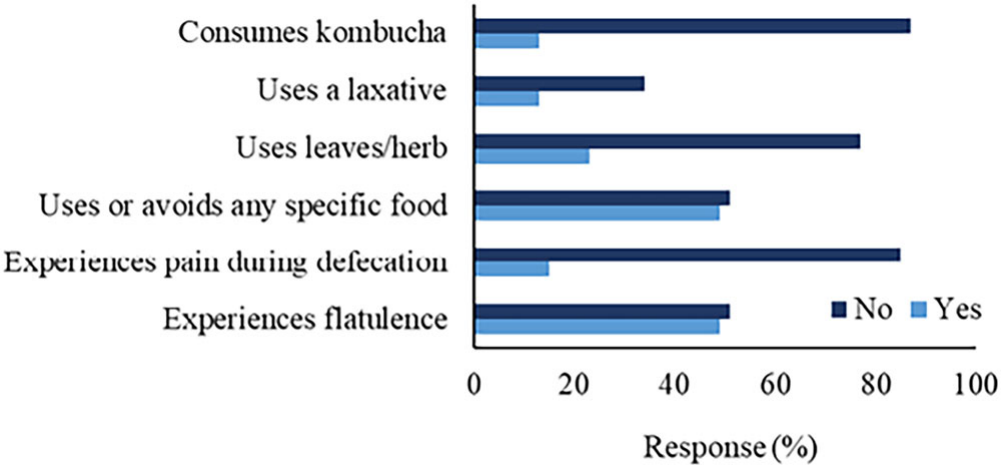
Classification of stool consistency according to the Bristol Stool Scale among participants who consumed active kombucha and control kombucha (sterilized). The *x*‐axis represents time in days, and the *y*‐axis represents the count of stool types. Values were compared using the Friedman test, followed by Dunn's test (*p* < 0.05). A significant difference was observed between active and control kombucha after 28 days of consumption.

Figure 3 shows the evolution of stool consistency, classified according to the Bristol Stool Scale, in participants who consumed active kombucha or control kombucha (autoclaved), assessed at baseline and after 14 and 28 days of consumption. This scale allows characterization of stool shape and texture, ranging from type 1 (hard, pellet‐like stools) to type 7 (entirely liquid stools), with types 3–5 considered indicative of ideal intestinal transit and good digestive health.

In the group that consumed active kombucha, a significant reduction in type 1 stools (separate and hard, difficult to pass) was observed between the start and day 28 (*p* = 0.022), along with a marked decrease in type 2 stools (sausage‐shaped but lumpy; *p* = 0.015). Conversely, there was a significant increase in type 4 stools (sausage‐shaped, smooth, and soft; *p* = 0.014), considered ideal in terms of consistency and ease of passage. No statistical differences were observed for types 3, 5, 6, and 7, although the overall pattern indicates a trend toward normalization of stool consistency over the consumption period. It is important to note that other uncontrolled factors in this study, such as dietary habits, physical activity level, hydration, and medication use, may have influenced the reported bowel patterns, contributing to individual variability and the lack of consistent effects in the other stool types (Lewis and Heaton 1997).

In the control kombucha group (autoclaved), no significant differences were detected between the evaluation time points, and no consistent improvement trend was observed. Comparison between groups showed a significant difference in type 2 stools after 28 days (*p* = 0.012), with lower scores in the control group.

These findings suggest that daily consumption of active kombucha for 4 weeks was associated with a shift toward normal stool consistency, with a reduction in the harder types and an increase in stools considered normal (types 4 and 5), suggesting a positive effect on intestinal motility and colonic microbiota balance (Villarreal‐Soto et al. 2018).

The observed pattern is consistent with recent clinical studies reporting improvements in intestinal transit and bowel movement perception following the consumption of kombucha or fermented beverages containing viable microorganisms. A randomized clinical trial (Isakov et al. 2023) observed a significant increase in the Bristol Stool Scale score (from 3.0 to 4.4; *p* = 0.001) and higher bowel movement frequency in women with constipation‐predominant irritable bowel syndrome after 10 days of consuming kombucha enriched with inulin.

Similarly, Fraiz et al. (2024) reported that the consumption of green tea–based kombucha during a weight loss intervention reduced gastrointestinal symptoms related to motility and promoted greater abdominal comfort, even without drastic changes in the microbiota. Although the number of clinical studies is still limited, the evidence converges on benefits for stool consistency, the sensation of incomplete evacuation, and the regulation of intestinal transit, which are attributed to the combined action of organic acids, enzymes, and live microorganisms present in the beverage.

In the present study, the absence of significant changes in the control group reinforces that the biological activity depends on the integrity of the symbiotic cultures, since autoclaved kombucha contains heat‐stable compounds but lacks viable microorganisms and active fermentation metabolites. This difference is consistent with the hypothesis that the functional effect of kombucha is mediated by the interaction between live bacteria and yeasts and the intestinal ecosystem, modulating colonic fermentation, hydration, and short‐chain fatty acid formation (Prajapati et al. 2024).

#### 3.6.3 IBS‐SSS Scale

Table 2 presents the percentage distribution of responses related to the IBS‐SSS before and after the consumption of active and control kombucha, allowing for a comparison of the evolution of gastrointestinal symptoms between the groups. The results suggest a noticeable reduction in abdominal pain after the consumption of active kombucha, with a 26.1% increase in “no pain” responses and a decrease of up to 60% in intermediate discomfort categories. This improvement may be related to modulation of the gut microbiota and the production of bioactive metabolites resulting from fermentation, such as organic acids and polyphenols, which exert anti‐inflammatory effects and regulate intestinal motility.

**TABLE 2:** Percentage distribution of responses related to the Irritable Bowel Syndrome Severity Scoring System (IBS‐SSS) before and after the consumption of active and control kombucha.

	Do you regularly suffer from abdominal pain?													
	None			Mild			Moderate			Severe			Very severe	
	Initial	Final	Variation (%)	Initial	Final	Variation (%)	Initial	Final	Variation (%)	Initial	Final	Variation (%)	Initial	Final
Active k	23	29	26.1	4	2	50.0	5	2	60.0	7	6	14.3	0	0
Control	29	32	13.8	3	3	0	7	4	42.9	0	0	0	0	0
	Do you frequently suffer from abdominal distension?													
Active k	22	27	22.7	5	4	20.0	4	2	50.0	8	6	25.0	0	0
Control	21	24	14.3	13	11	15.4	5	4	20.0	0	0	0	0	0

	Are you satisfied with your bowel movements?											
	Very dissatisfied			Dissatisfied			Satisfied			Very satisfied		
	Initial	Final	Variation (%)	Initial	Final	Variation (%)	Initial	Final	Variation (%)	Initial	Final	Variation (%)
Active k	2	1	50.0	9	6	33.3	21	17	19.0	7	16	128.5
Control	2	3	50.0	15	5	66.8	15	30	10.0	7	1	85.7

	To what extent does the functioning of your bowel affect or interfere with your life in general?											
	Not at all			A little			Quite a lot			Completely		
	Initial	Final	Variation (%)	Initial	Final	Variation (%)	Initial	Final	Variation (%)	Initial	Final	Variation (%)
Active k	0	0	0	15	11	26.7	15	18	20.0	9	10	11.1
Control	0	0	0	9	7	22.2	19	21	10.5	11	10	9.1

Recent evidence confirms that regular consumption of kombucha can alleviate gastrointestinal and constipation symptoms in individuals with irritable bowel syndrome, possibly by restoring microbial balance and reducing local inflammatory processes (Fraiz et al. 2024). In contrast, the autoclaved kombucha showed a more modest improvement, suggesting that part of the beneficial effect depends on microbial viability and the symbiotic interaction of live cultures with the host, rather than solely on the presence of heat‐stable compounds.

Active kombucha also showed a consistent reduction in episodes of abdominal bloating, with a 22.7% increase in “no symptoms” responses and a 50% decrease in “moderate to severe” responses. This result is consistent with studies demonstrating that fermented beverages containing viable microorganisms promote balanced colonic fermentation, reducing excessive gas formation and improving the digestion of fibers and fermentable carbohydrates (Soares et al. 2023). In contrast, the control kombucha showed only modest changes, suggesting that the effects observed in the active kombucha are primarily driven by the microbial and metabolic activity of the symbiotic cultures (Cavicchia and Almeida 2022), while the autoclaved version retains only stable compounds with lower functional impact.

The most pronounced improvement was observed in satisfaction with bowel movements. In the active kombucha group, there was a marked increase in the proportion of participants reporting being “very satisfied” (an increase of 128.5%), accompanied by a reduction in the “dissatisfied” category. These results indicate a noticeable improvement in intestinal comfort and regularity, possibly associated with the combined action of organic acids (acetic, gluconic, and glucuronic) and the symbiotic activity of yeasts and lactic acid bacteria, which stimulate intestinal motility and microbial homeostasis.

Recent clinical trials report similar results: in a randomized study with inulin‐enriched kombucha, a significant increase in bowel movement frequency and a reduction in the sensation of straining were observed after 10 days of consumption (Isakov et al. 2023). The control group showed the opposite trend, with an increase in “very dissatisfied” responses and a marked decrease in overall satisfaction, reinforcing that the presence of live cultures is crucial for eliciting a positive physiological response.

Regarding the impact on quality of life, the results showed a slight improvement among active kombucha consumers, with a small decrease in “not much” responses and an increase in “quite a lot,” suggesting reduced interference of gastrointestinal symptoms in daily activities. The observed pattern is consistent with studies indicating that live kombucha may contribute to gastrointestinal well‐being, possibly through anti‐inflammatory and immunomodulatory mechanisms mediated by the microbiota (Ecklu‐Mensah et al. 2024; Soares et al. 2023). In the control group, the changes were smaller, supporting the hypothesis that the biological activity depends on the integrity of the fermentative cultures.

Slow intestinal transit favors the fermentation of food residues by gut bacteria, increasing gas production and exacerbating bloating (Franca et al. 2021). Factors such as a low‐fiber diet, inadequate fluid intake, and altered intestinal motility can contribute to this condition. Management of abdominal bloating in individuals with constipation involves strategies such as dietary adjustments, increased fiber and fluid intake, regular physical activity, and, in some cases, the use of probiotics and mild laxatives, always under professional guidance (Souza et al. 2022).

In a study conducted by Isakov et al. (2023), the efficacy of a specialized kombucha‐based food product, enriched with inulin, was investigated in the treatment of constipation‐predominant irritable bowel syndrome (IBS‐C). The results indicated significant improvements in bowel movement frequency, stool consistency, and participants’ quality of life. These findings suggest that the functional beverage may represent a promising dietary strategy for improving self‐reported gastrointestinal symptoms and intestinal comfort. However, it is important to emphasize that the participants included in this study were not clinically diagnosed with IBS. Therefore, the IBS‐SSS questionnaire was used only as a validated tool for monitoring the evolution of gastrointestinal symptoms and intestinal discomfort throughout the intervention period, rather than for evaluating therapeutic effects in diagnosed IBS patients.

Although some participants in the control group also reported perceived improvements, possibly due to placebo‐related effects or the presence of stable fermentation‐derived compounds, the active kombucha showed a more consistent pattern of improvement in self‐reported gastrointestinal symptoms and intestinal function. These findings suggest that the presence of viable microbial communities and fermentation‐derived metabolites may have contributed to the observed improvements in intestinal function and overall gastrointestinal well‐being.

### 3.7 Limitations and Future Perspectives

Some limitations of the present study should be acknowledged. The microbial composition of the SCOBY was not identified at the strain level prior to fermentation, which limits more specific interpretations regarding probiotic functionality and strain‐dependent effects. In addition, although the microbiological analyses confirmed the presence and maintenance of viable microbial populations during storage, no molecular characterization of the microbial consortium was performed.

The clinical trial involved a relatively small convenience sample of generally healthy individuals with self‐reported gastrointestinal symptoms rather than participants formally diagnosed with IBS. Therefore, the observed clinical improvements should be interpreted as preliminary findings and should not be extrapolated to broader populations or to patients with clinically diagnosed gastrointestinal disorders.

Another limitation of the present study is that microbiological challenge tests against relevant foodborne pathogens, such as *Listeria monocytogenes*, *Salmonella* spp., and *Escherichia coli* O157:H7, were not performed. Although the low pH and acidic environment of kombucha are recognized as important barriers to pathogen survival, challenge studies are still required to validate the microbiological safety and industrial robustness of the beverage under commercial production and storage conditions. Such investigations represent an important direction for future studies.

Future investigations should include microbial strain identification at the species and strain levels, microbiological challenge studies using relevant foodborne pathogens, and longer clinical intervention periods to further elucidate the mechanisms underlying the functional effects and microbiological safety of blueberry‐enriched kombucha.

## 4 Conclusion

The development of blueberry‐flavored kombucha resulted in a promising functional beverage, combining favorable microbiological, physicochemical, and bioactive characteristics. The formulation maintained the viability of the characteristic kombucha microbiota and demonstrated stability in terms of pH and acidity, in addition to exhibiting substantial antioxidant activity attributed to the presence of phenolic compounds and flavonoids, with particular emphasis on the preservation of anthocyanins in the active formulation.

The clinical study suggested beneficial effects associated with 4 weeks of daily consumption of the beverage, particularly improvements in stool consistency, reductions in self‐reported abdominal symptoms, and a more favorable perception of intestinal function compared with the control group. These findings should be interpreted as preliminary evidence and warrant confirmation in larger clinical trials involving clinically characterized populations.

## Nomenclature

ABTS: 2,2′‐azino‐bis(3‐ethylbenzothiazoline‐6‐sulfonic acid)
ANOVA: analysis of variance
AOAC: Association of Official Analytical Chemists
BMI: body mass index
CFU: colony forming units
CIELAB: Commission Internationale de l'Éclairage Lab* Color System
DPPH: 2,2‐diphenyl‐1‐picrylhydrazyl
GAE: gallic acid equivalents
IBS‐SSS: Irritable Bowel Syndrome Severity Scoring System
IC_50_
: half maximal inhibitory concentration
MRS: de Man, Rogosa, and Sharpe (culture medium)
NaClO: sodium hypochlorite
PET: polyethylene terephthalate
QE: quercetin equivalents
SCOBY: symbiotic culture of bacteria and yeasts

## Author Contributions

**Angelica Morgan Anselmini**: methodology, formal analysis, data curation, investigation. **Gabrieli Lorandi**: investigation, formal analysis, methodology. **Marcieli Peruzzolo**: methodology, formal analysis, investigation. **Itamar Luís Gonçalves**: validation, visualization. **Rogério Luis Cansian**: methodology, writing – original draft. **Jamile Zeni**: writing – original draft, investigation. **Eunice Valduga**: data curation, formal analysis. **André Keng Wei Hsu**: conceptualization, writing – original draft. **Vivian Polachini Skzypek Zanardo**: conceptualization, writing – review and editing, supervision, resources. **Geciane Toniazzo Backes**: visualization, project administration, resources, supervision, writing – review and editing, conceptualization.

## Conflicts of Interest

The authors declare no conflicts of interest.
